# *Saraca indica* Bark Extract Shows *In Vitro* Antioxidant, Antibreast Cancer Activity and Does Not Exhibit Toxicological Effects

**DOI:** 10.1155/2015/205360

**Published:** 2015-03-16

**Authors:** Navneet Kumar Yadav, Karan Singh Saini, Zakir Hossain, Ankur Omer, Chetan Sharma, Jiaur R. Gayen, Poonam Singh, K. R. Arya, R. K. Singh

**Affiliations:** ^1^Division of Toxicology, CSIR-Central Drug Research Institute, BS-10/1, Sector 10, Jankipuram Extension, Sitapur Road, P.O. Box 173, Lucknow 226031, India; ^2^Division of Endocrinology, CSIR-Central Drug Research Institute, BS-10/1, Sector 10, Jankipuram Extension, Sitapur Road, P.O. Box 173, Lucknow 226031, India; ^3^Division of Pharmacokinetics & Metabolism, CSIR-Central Drug Research Institute, BS-10/1, Sector 10, Jankipuram Extension, Sitapur Road, P.O. Box 173, Lucknow 226031, India; ^4^Academy of Scientific and Innovative Research, New Delhi 110 001, India; ^5^Division of Botany, CSIR-Central Drug Research Institute, BS-10/1, Sector 10, Jankipuram Extension, Sitapur Road, P.O. Box 173, Lucknow 226031, India

## Abstract

Medicinal plants are used as a complementary and alternative medicine in treatment of various diseases including cancer worldwide, because of their ease of accessibility and cost effectiveness. Multicomposed mixture of compounds present in a plant extract has synergistic activity, increases the therapeutic potential many folds, compensates toxicity, and increases bioavailability. *Saraca indica* (family Caesalpiniaceae) is one of the most ancient sacred plants with medicinal properties, exhibiting a number of pharmacological effects. Antioxidant, antibreast cancer activity and toxicological evaluation of *Saraca indica bark extract* (SIE) were carried out in the present study. The results of the study indicated that this herbal preparation has antioxidant and antibreast cancer activity. Toxicological studies suggest that SIE is safer to use and may have a potential to be used as complementary and alternative medicine for breast cancer therapy.

## 1. Introduction

In recent years, large numbers of research studies are conducted, which stabilised the therapeutic use of antioxidants in treatment of various diseases such as cardiovascular diseases, diabetes, neurodegeneration, inflammation, and cancer [1–3]. Free radicals like hydroxyl, peroxyl, and superoxide radicals can be produced during normal metabolic function, are very transient and highly reactive, cause damage to the biomolecules, leading to adverse effects on human health, and cause severe diseases [4, 5].

Several studies showed that elevated level of free radicals is associated with carcinogenesis [6–9]. ROS is a double edge sword while ROS generation is essential for cell survival, proliferation, and progression of cancer cells. In contrast increased level of ROS also induces the apoptosis and hence plays a crucial role in cancer chemotherapy [10–12]. Damage caused by free radicals can result in formation of single and double strand breaks of DNA and oxidation of purine and pyrimidine bases, leading to genome instability and subsequent carcinogenesis [13–15]. Therefore, protection of cell from oxidative damage by antioxidant supplements is very helpful in prevention and treatment of cancer [16–18].

Since ancient time, medicinal plants were used as key therapeutic agents all over the globe, especially among the rural communities of developing countries due to the unavailability of an accessible and affordable primary health care system [19, 20]. According to World Health Organization (WHO), 80% people across the globe used medicinal plants. A wide range of biological and pharmacological properties of medicinal plants manifest their therapeutic potential, for the treatment of various diseases [21–23].

*Saraca indica* (family Caesalpiniaceae) also known as* Saraca asoca* is one of the most ancient sacred plants widely distributed throughout the Indian subcontinent [24, 25]. Various medicinal uses of* Saraca indica* had been reported in Charaka Samhita (100 A.D.) [26]. Different parts of the plant exhibit a number pharmacological effects like antihyperglycemic, antipyretic, antibacterial, anthelmintic, activity, and so forth, which are well described in literature [27–30]. A traditional drug Asoka Aristha used for the treatment of menorrhagia is originated from* Saraca indica* [31]. Secondary metabolites like flavonoids, terpenoid, lignin, phenolic compounds, tannins, and so forth are reported from* Saraca indica* stem bark extracts and found responsible for their therapeutic action [32–38].

Cancer is responsible for the majority of the death all over the world, out of which breast cancer is the most commonly occurring cancer in women. It is estimated that approximately 25% of all cancers diagnosed in women cause 0.52 million deaths worldwide, out of which approximately 62.13% of deaths occur in less developed regions of the world [39, 40].

Surgery, radiation and chemotherapy are the standard methods for the treatment of cancer including breast cancer [41]. These therapies showed success to a varying extent to give relief from symptoms and enhance the survival time of patients; but they are also associated with severe side effects, as in case of chemotherapy drugs, they have very narrow therapeutic indexes in terms of nonselective toxic effects on normal tissues and they are also associated with many unwanted side effects such as nausea, vomiting, anaemia, loss of hair, pain in joints, lymphedema, and even the development of secondary cancers [41–45].

Uses of herbal medicine in the treatment of breast cancer and other types of cancers are well substantiated in the literature [46–50]. Hartwell (1982) described more than 3000 medicinal plants, possessing anticancer properties and subsequently used as potent anticancer drugs [51–53]. Medicinal plants have the ability to provide accessible, cost effective, and also a relatively safe treatment, in comparison to the standard method [43, 45, 50]. Although medicinal plants are considered nontoxic, a number of safety studies reported that they can cause various side effects; hence safety evaluation of medicinal plants is also required [54, 55].

The present study was carried out to evaluate antioxidant and anticancer activity of* Saraca indica* bark extract (SIE) in breast cancer cell lines (MCF-7 and MDA-MB-231).* In vivo* repeated dose toxicity study was conducted to evaluate the safety of the oral administration of SIE. Results from this study will be helpful to understand the use of* Saraca indica* stem bark extracts in prevention and treatment of cancer as well as to evaluate any adverse effects associated with use of SIE for health benefits.

## 2. Materials and Methods

*Ethics Statement*. All animal procedures have been approved and prior permission from the Institutional Animal Ethical Committee was obtained as per the prescribed guidelines (IAEC Approval No. IAEC/2012/86).

### 2.1. Plant Material

*Saraca indica* bark was collected and the sample was authenticated by Dr. K. R. Arya, Principal Scientist, Botany Division, CSIR-Central Drug Research Institute Lucknow (U.P.), India. Specimen sample of* Saraca indica* has been allotted a voucher sample specimen No. KRA/23998 and kept at the medicinal plant repository of the institute.

### 2.2. Preparation of* Saraca indica* Bark Extracts (SIE)

The* Saraca indica* Bark was dried in an oven at 40°C for 5 days and then grounded in an electric blender. The powder was suspended in 80% alcohol and left at room temperature for 24 h. The crude extract was filtered using 125 mm Whatman qualitative filter paper under sterile condition. This process was repeated 5 times and then the solvent (alcoholic extract of* Saraca indica* Bark), thus collected, was evaporated to dryness under reduced pressure using a rotary evaporator below 50°C. The residue was further subjected to dryness by incubating them for 8 days at 37°C. The extract was kept at 4°C until use. The yield of the extract was 9.7% (w/w).

### 2.3. Determination of Total Phenolic Contents in the Plant Extracts

The concentration of phenolic compounds in SIE was determined by spectrophotometric method. Methanolic solution of the extract in the concentration of 1 mg/mL was used in the analysis. Briefly the reaction mixture was obtained by mixing 0.5 mL of methanolic solution of extract, 2.5 mL of 10% Folin-Ciocalteu's reagent dissolved in water, and 2.5 mL 7.5% NaHCO_3_. Blank was solution containing 0.5 mL methanol, 2.5 mL 10% Folin-Ciocalteu's reagent dissolved in water, and 2.5 mL of 7.5% of NaHCO_3_ and absorbance was determined using spectrophotometer at *λ*_max⁡_ = 765 nm. Same method was used for solution of gallic acid and the calibration line was drawn. All experiment was performed in triplet. Phenolic content of extract was expressed in terms of gallic acid equivalent (mg of GA/g of extract) [56].

### 2.4. *In Vitro* Antioxidant Activity

#### 2.4.1. DPPH Radical Scavenging Assay

The antioxidant activity of the SIE was measured on the basis of free radical scavenging activity of plant extract. SIE or standard was added to 200 *μ*L of DPPH in methanol solution in a 96-well microtitre plate. Mixtures were incubated at 37°C for 30 min and then absorbance of mixtures was determined at 490 nm. Blank readings were taken to calculate the remaining DPPH and IC_50_ value was determined [57].

#### 2.4.2. Nitric Oxide Free Radical Scavenging Activity

To measure the nitric oxide free radical scavenging activity, 50 *μ*L of plant extract of different concentrations, dissolved in DMSO, was taken and then methanol was added to make the volume 150 *μ*L. 2.0 mL of sodium nitroprusside (10 mM) in phosphate buffer saline was added in each tube and they were incubated at room temperature for 150 min. After the incubation, 5 mL of Griess reagent was added to each tube and the absorbance of chromophore formed was measured at 546 nm on spectrophotometer. Same procedure was repeated with ascorbic acid (positive control) and methanol (blank which served as control) [58, 59]. The IC_50_ values of plant extract and ascorbic acid were calculated as

(1) %ScavengingReduction =Absorbance  of  controlAbsorbance  of  test  sampleAbsorbance  of  control   −Absorbance  of  test  sampleAbsorbance  of  control×100.

#### 2.4.3. Lipid Peroxidation Inhibition Activity

MDA assay was used to determine the lipid peroxidation inhibition effect of SIE as described by Baharum et al. [60]. Briefly rat liver tissue (2.0 g) was sliced and homogenized in 10 mL 15 mM KCl–Tris-HCl buffer (pH 7.2). The reaction solution (0.25 mL liver homogenate, 0.1 mL Tris-HCl buffer (pH 7.2), 0.05 mL 1 mM ascorbic acid, 0.05 mL 4 mM FeCl_2_) and 0.05 mL of plant extract was taken in tube. The reaction tube was incubated at 37°C for 1 h. After incubation 0.5 mL 0.1 N HCl, 0.2 mL 9.8% sodium dodecyl sulfate, 0.9 mL distilled water, and 2 mL 0.6% TBA were added to each tube and vigorously shaken. Then, the tubes were placed in a boiling water bath at 100°C for 30 min. After cooling, the flocculent precipitate was removed by adding 5 mL *n*-butanol, mixed well, and centrifuged at 9,000 rpm for 10 min. The absorbance (Abs) of the supernatant was measured at 532 nm [61]. The percentage of lipid peroxidation inhibition was measured using the following equation:

(2) Lipid  peroxidation  inhibition(%)   =Abs  control−Abs  sampleAbs  control×100%.

### 2.5. Test Animals

CF rats (150–175 gm) were obtained from the National Laboratory Animal Center (NLAC), Central Drug Research Institute, Lucknow, India. The animals were housed in polycarbonate cages with bedding at 25 ± 2°C temperature and 30–60% relative humidity with a 12 h light and dark cycle throughout the study period. CF Rats were allowed to acclimatize at experimental room conditions for 7 days prior to toxicity study. The animals were fed a standard rodent pellet diet and water* ad libitum* [62–66].

### 2.6. Toxicity Study

Healthy CF rats were randomly divided into five groups, with 5 animals per group. One group served as the control and received 1% gum acacia in distilled water. Four other groups were orally treated by gavage with different doses of SIE (500, 1000, 1500, and 2000 mg/Kg B.Wt.) suspended in water with 1% gum acacia. Toxicity study was carried out as recommended by toxicity evaluation guideline of Schedule Y [67].

Rats were observed for toxicity symptoms as defined in the Common Toxicity Criteria developed by the Cancer Therapy Evaluation Program with some modification if needed (National Cancer Institute, 1999, Common Toxicity Criteria Version 2.0, Cancer Therapy Evaluation Program). Their body weight changes and food and water intake were recorded on alternate days.

At the end of the study, the animals were fasted overnight, although water was made available* ad libitum.* They were then anesthetized using diethyl ether for necropsy and blood collection. Blood was collected in two different tubes: one tube containing the anticoagulant EDTA and one tube without anticoagulant for hematological and biochemical examination, respectively. The vital organs of animals were dissected and removed with care. Weight of each organ was taken and examined for macroscopic features.

### 2.7. Hematological and Biochemical Analysis of Blood

Blood collected in EDTA coated vials was analyzed using MS-9 automatic hematology analyzer (Melet Schloesing Ltd., France), shortly after its collection. Blood samples were collected for serum chemistry analysis in tubes lacking anticoagulant and placed at room temperature for at least 90 min prior to centrifugation; after centrifugation at 1600 g for 10 min, serum was collected and biochemical parameters were measured using fully automated random access clinical chemistry analyzer (Beckman Synchron CX5, USA).

### 2.8. Cell Culture and Reagents

Breast cancer cell lines, MDA-MB-231, MCF-7, and normal human cell line HEK-293 were maintained in DMEM supplemented with 10% fetal bovine serum (GIBCO BRL Laboratories, New York, USA) and 1% penicillin-streptomycin solution (Sigma Chemical Co., St. Louis, MO, USA) in humid environment at 37°C with 5% CO_2_.

### 2.9. Cell Proliferation Inhibition Assay

Antiproliferative property of SIE against breast cancer cells (MCF-7, MDA-MB-231) was evaluated by MTT assay and safety evaluation was done in normal human cell (HEK-293). Briefly, cells (1 × 10^4^/well) were seeded in 96-well plate. After 24 h of growth, cells were treated with different concentration of SIE for 24 h, 48 h, and 72 h. At the end of incubation, 20 *μ*L of MTT (5 mg/mL) was added in each well and incubated for 3 h, media at the end of incubation media along with MTT were removed and formazan crystals were dissolved in 200 *μ*L dimethyl sulfoxide. The absorbance was recorded at 540 nm by ELISA plate reader. IC_50_ was determined using Graphpad Prism3 version software.

### 2.10. Cell Cycle Analysis

Distribution of cells in different phases of cell cycle following treatment was analyzed by flow cytometer using propidium iodide (PI) staining. MCF-7 cells (1 × 10^6^) were seeded in T-25 culture flasks. After 24 h of growth, cells were treated with different concentration of SIE for 72 h. At the end of incubation, all cells including floating cells were harvested. Cells were fixed in ice cold 70% ethanol at 4°C for 1 h. Following incubation cells were pellet down and resuspended in PBS containing PI (30 *μ*g/sample) and RNAse A (30 *μ*g/sample) and incubated for 30 min at room temperature in dark [68]. Samples were acquired by BD FACS Calibur flow cytometer and analysed by using a software BD FACSuite Software.

### 2.11. Apoptosis Analysis

Apoptosis induced by SIE was measured by Annexin-V-FITC-PI staining using flow cytometer. MCF-7 cells (1 × 10^6^/well) were seeded in 6-well plate and allowed to grow for 24 h. Cells were treated with different concentration of SIE for 72 h. At the end of the treatment, all cells including floating cells were harvested, washed with PBS, and stained with Annexin-V-FITC and propidium iodide (Sigma-Aldrich) for 10 min at RT [68, 69]. Samples were acquired by flow cytometer FACS caliber (BD biosciences).

### 2.12. Microscopic Analysis by Hoechst Staining

Morphological changes in the nucleus induced by SIE treatment were studied by Hoechst 33258 staining. MCF-7 cells (2 × 10^4^/well) were seeded in 24-well plate and after 24 h of growth, cells were treated with different concentration of SIE for 72 h and cells were fixed with 4% paraformaldehyde for 10 min and then washed with PBS and permeabilised with 3% paraformaldehyde containing 0.5% triton X-100 for 30 min and then stained with Hoechst 33258 stain (Invitrogen 3 mg/mL) for 30 min and images were captured by Microscope (Leica).

### 2.13. Chemical Analysis by Mass Spectrometry

For chemical characterization mass spectrometric detection was performed on API 4000 Q TRAP mass spectrometer (AB Sciex Toronto, Canada) equipped with an electrospray ionization (ESI) source. The SIE was dissolved in 50 : 50 solution of A: 10 mM ammonium acetate, 0.1% formic acid in water, and B: 50 : 50 ACN : MeOH and infused with Harvard Infusion Pump 11 (Harvard Apparatus, USA) with optimised flow rate of 20 *μ*L/minute.

The extract was scanned both in positive and negative ion mode within a range of 100 to 800* m/z*, where the positive ion mode showed greater ionization and sensitivity. Data profiling was recorded at a speed of 0.15 s/scan and the scanning delay of 0.01 s during analysis. The main working parameters of the mass spectrometer were (i) ion spray voltage (ISV)-5500, (ii) curtain gas (CUR)-25, and (iii) ion source gas one (GS1) and two (GS2)-10 and quadruple set on unit resolution. Data processing was performed using Analyst version 1.5 software package (SCIEX).

### 2.14. Statistical Analysis

The data generated during the study was analyzed using one-way ANOVA test and the *P* value less than 0.05 was considered to be significant.

## 3. Results

### 3.1. Total Phenolic Contents

Phenolic phytocompounds of plants show powerful free radical scavengers activity. They have potential to inhibit the lipid peroxidation by neutralizing peroxyl radicals generated during the oxidation of lipids [70]. The total phenolic content of SIE accessed using the Folin-Ciocalteu's reagent is expressed in terms of gallic acid equivalent. The values obtained for the concentration of total phenols are expressed as mg of GA/g of extract. The total phenolic content of SIE was calculated to be 55 mg GA/g.

### 3.2. Antioxidant Activity

#### 3.2.1. DPPH Radical Scavenging Assay

The antioxidant activity of SIE was evaluated using the DPPH free radical scavenging method. Ascorbic acid was used as standard compound. The SIE exhibited strong antioxidant activity in the DPPH inhibition assay as evidenced by the low IC_50_ values. The IC_50_ value obtained was 38.5 *μ*g/mL in the DPPH inhibition assays.

#### 3.2.2. Nitric Oxide Scavenging Activity

Nitric oxide scavenging activity was performed with SIE using ascorbic acid as standard compound. In this study it was observed that SIE has ability to scavenge nitric oxide radical in dose dependent manner. The IC_50_ value of SIE was found to be 29.1 *μ*g/mL in nitric oxide radical scavenging assay.

#### 3.2.3. Lipid Peroxidation Inhibition Activity

Lipid peroxidation inhibition activity was measured* in vitro* by determining the malondialdehyde (MDA) and related compounds in rat liver homogenate [71]. Lipid peroxidation is one of the reasons of occurrence of various diseases including cancer [72]. So, inhibition of lipid peroxidation is an indicator of therapeutic property of plant extract. The SIE exhibited lipid peroxidation inhibition activity and the IC_50_ value was 66 *μ*g/mL.

### 3.3. Repeated Dose Toxicity Study

#### 3.3.1. General Observations

**Figure 1:**
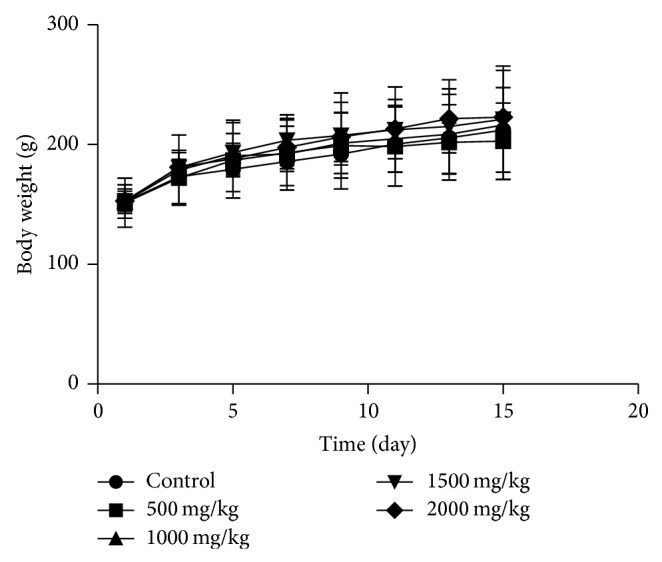
The changes of body weight after oral administration of SIE for in male rats. Data were analyzed by one-way analysis of variance. There was no significant difference between control and test groups.

**Figure 2:**
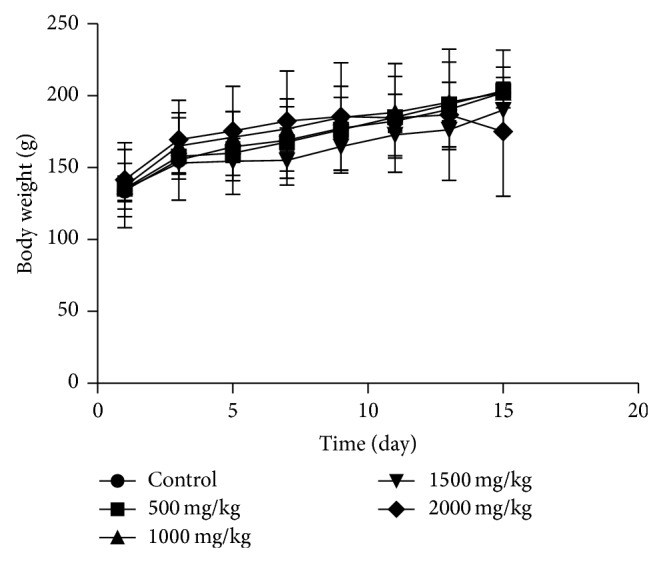
The changes of body weight after oral administration of SIE for in female rats. Data were analyzed by one-way analysis of variance. There was no significant difference between control and test groups.

The effects of oral administration of SIE are summarized in Table 1. The results showed that oral administration of SIE 2000 mg/Kg B.Wt. does not produce any sign of toxicity in both sex. There was no significant difference in body weight of control and treated groups in both sexes (Figures 1 and 2).

#### 3.3.2. Biochemical and Hematological Analysis

The effect of SIE on biochemical and hematological parameters was summarized in Tables 2, 3, 4, and 5. Statistical analysis of the results shows that the SIE does not produce any sign of toxicity. Biochemical parameters which include markers of hepatotoxicity (ALT, AST, ALP, and TBIL) and nephrotoxicity (CREA and BUN) indicate nontoxic effects of SIE on liver and kidney. Blood parameters were statistically similar in control and treated groups, and any shape related abnormalities in RBCs were not observed.

Macroscopic analysis of major vital organs did not show any significant change in colour texture and size when compared with the control in male and female. Reproductive organ weight does not show any significant difference between control and treated groups in case of both sexes (Tables 6 and 7).

### 3.4. Anticancer Activity of* Saraca indica* Extract

**Figure 3:**
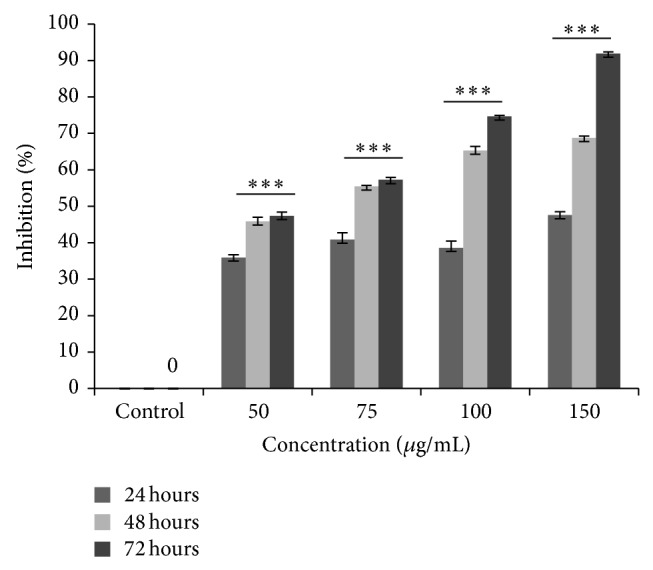
SIE induced MCF-7 cells inhibition. MCF-7 cells (1 × 10^4^ cells/well) were seeded in 96 well culture plates and after 24 h of growth cells were treated with different concentrations of SIE for different time point and percent cells inhibition was measured by using MTT assay and data represented in ±SE and statistatical significance determined as compared to control by using one way Anova.

The antiproliferative activity of SIE was evaluated in different breast cancer cells (MDA-MB-231, MCF-7). SIE inhibited proliferation of MCF-7 (ER positive) cells and MDA-MB-231 (ER negative) cells but its activity was more prominent in MCF-7 cells with IC_50_ 73.6 ± 0.625 *μ*g/mL and 128 ± 0.914 *μ*g/mL in MCF-7 and MDA-MB-231 cells, respectively (Table 8). SIE inhibit the proliferation of MCF-7 cells in dose as well as time dependently at 48 h and 72 h but dose dependency was not seen at 24 h (Figure 3).* In vitro* safety evaluation was done in HEK-293 cells and the SIE does not induce significant cytotoxicity up to the concentration of 200 *μ*g/mL (Table 8).

**Figure 4:**
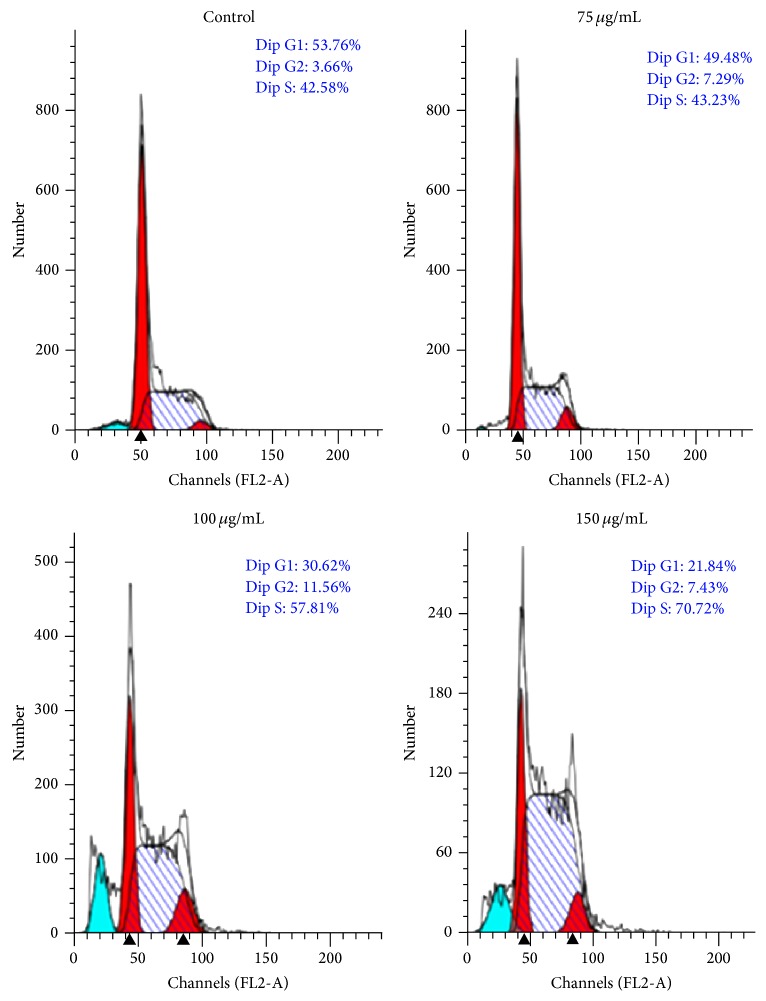
Effect of SIE on cell cycle in MCF-7 cells. 1 × 10^5^ Cells were seeded in T-25 flask and after 24 h cells were treated with 75 *µ*g/mL, 100 *µ*g/mL and 150 *µ*g/mL of SIE or vehicle control for 72 h, stained with propidium iodide (PI) and samples were acquired by flow-cytometer.

**Figure 5:**
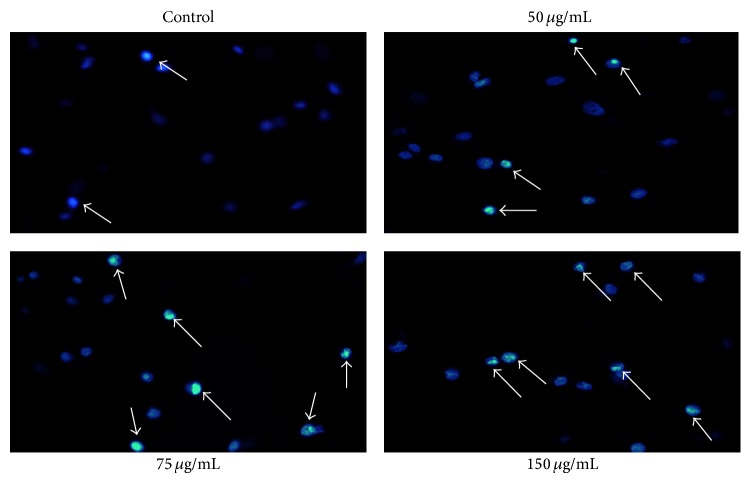
Induction of nuclear fragmentation by SIE in MCF-7 cells: 2 × 10^4^ cells/well were seeded in 24-well culture plate and allowed to grow for 24 h and then treated with different concentrations of SIE for 72 hours and stained with Hoechst 33258 stain following standard protocol and image was captured by Leica Microscope at 20x magnification.

**Figure 6:**
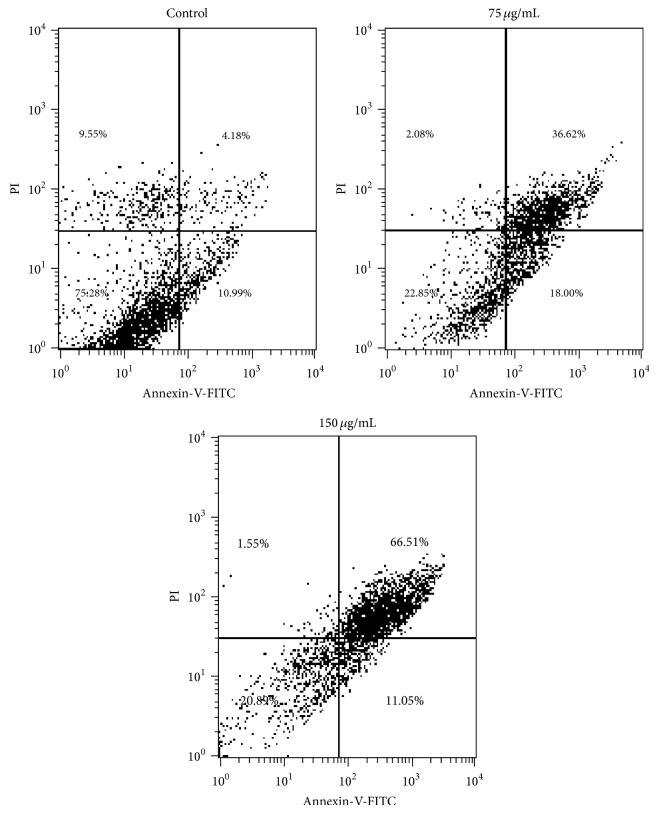
Induction of apoptosis by SIE in MCF-7 cells. 10 × 10^5^ cells/well were seeded in 6 well plates after 24 h of growth cells were treated with 75 *µ*g/mL and 150 *µ*g/mL of SIE and stained with Annexin V-FITC –PI and samples were acquired with flow cytometry.

Distribution of cells in different phases of cell cycle followed by SIE treatment in MCF-7 cells for 72 h, cell cycle analysis was carried out using propidium iodide (PI) staining by flow cytometry. SIE treatment arrested cells at S phase of cell cycle (Figure 4) probably by interfering with DNA replication [73]. Furthermore, morphological changes in the nucleus induced by SIE were studied with Hoechst 33258 staining, a popular nuclear counter stain that emits blue fluorescence when bound to two dsDNA, which stain nucleus of the live cells with uniform blue fluorescence while apoptotic cells had bright blue nuclei due to karyopyknosis and chromatin condensation [74]. Our results showed increase in fluorescence and chromatin condensation in MCF-7 cells followed treatment with SIE as compared to vehicle control in dose dependent manner (Figure 5). We also confirmed if the inhibition of cell growth induced by SIE is associated with physiological apoptosis (programmed cell death) or nonspecific necrosis. We stained the MCF-7 cells with Annexin-V-FITC-PI followed by SIE treatment. Flow cytometric data showed that SIE induces significant increase in the late apoptotic cells population and induction of apoptosis dose dependently (Figure 6). These data indicate that SIE inhibit proliferation of MCF-7 cells by arresting cells in S-phase which ultimately induces programme cell death by apoptosis.

### 3.5. Chemical Characterization of SIE

**Figure 7:**
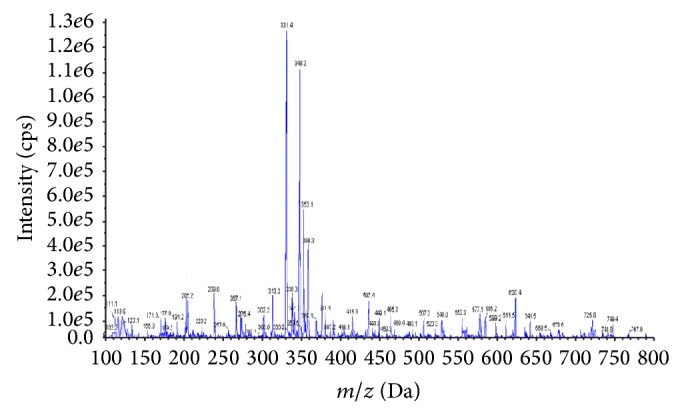
Mass fingerprinting chromatogram of SIE in positive ion (*M* + 1) mode.

Various components of different extracts of* Saraca indica* have been extensively reported as by Gahlaut et al. (2013), Kashima and Miyazawa (2012), Shirolkar et al. (2013), and Mittal et al. (2013) [26, 29, 75, 76]. The combined result of detected compounds from the mass spectrometric analysis and literature is shown in Table 9 (Figure 7).

## 4. Discussion

Various scientific studies show that aberrance in redox balance with elevated level of oxygen-free radicals, reactive oxygen species (ROS), and reactive nitrogen species (RNS) plays an important role in the origin and progression of most human diseases including cancer [77–81].

Reactive oxygen species (ROS) act as secondary messenger in intracellular signalling cascades and elevated level of ROS associated with carcinogenesis by promoting initiation, progression, and metastasis of cancer cells. It also induced DNA damage leading to genetic lesions that initiate tumorigenicity and subsequent tumor progression [8, 82–84]. However, many studies also suggested that free radicals are essential mediators of apoptotic pathway for triggering cell death and therefore function as anticancer agents. Thus, free radicals production approach is used in nonsurgical therapeutic methods for cancer therapy, including chemotherapy, radiotherapy, and photodynamic therapy [82, 85, 86]. Free radicals produced in cancer therapy are associated with serious side effects. Furthermore, elevated level of ROS in cancer cell leads to intercellular transfer of hydrogen peroxide (H_2_O_2_) to neighbouring cells, and stimulates them to acquire uncontrolled ROS production [83]. Free radical scavenger activity plays a protective role in normal healthy cells. They prevent the ROS from spreading and ultimately protect the adjacent cells from oxidative DNA damage and check the cancer progression. Many clinical trials have also suggested that intake of exogenous antioxidants can protect the healthy cells from oxidative stress as well as ameliorate toxic side effects of cancer therapy without affecting therapeutic efficacy [85].

Extracts of medicinal plants have been used for the treatment of various diseases, including cancer all over the globe, as they are easily prepared, standardized, and stored. Herbal extracts are also cost effective which increase their accessibility to the patients of all economic status [87, 88]. Global health policies promote the therapeutic use of herbal extract. World Health Organization (WHO) also encourages the use of medicinal plants in the treatment of disease [21, 89, 90].

Medicinal plants used as therapeutic agents are considered nontoxic for human consumption, while many studies reported the various side effects of medicinal plant [54, 55]. Medicinal plants uses for health benefit are not taken under the appropriate instruction and consultant of physician. Although people are using medicinal plants from ancient time, safety evaluation of these medicinal plants are required [49].

The modern approach to discover a new drug molecule involves either isolation from a natural source or the synthesis of a particular compound responsible for a therapeutic effect [46, 91, 92]. However, a complex interplay of various signalling pathways is responsible for carcinogenesis and cancer progression, which limit the efficacy of a single drug to provide a desired therapeutic result. As of now, inability of single drug to produce most effective results in breast cancer treatment enhances the future prospective of medicinal plants as complementary and alternative medicines in cancer therapy [93–95].

Extracts of medicinal plants are multicomposed mixtures of active components; they show their synergistic effect by acting at the same or different nodes of a cancer signalling network resulting in increase of therapeutic potential many folds, in comparison to a single drug-target therapy, and also compensate the toxicity and increased bioavailability of active compounds [96–101]. Ability to target the multiple nodes of cancer signalling network may restrict the cancerous cells to develop the resistance against medicinal plant extracts [102].

In present study, SIE showed growth inhibition in both ER positive (MCF-7) and ER negative (MDA-MB-231) breast cancer cells. SIE induced significant growth inhibition in MCF-7 cells as compared to MDA-MB-231 cells by inducing apoptosis mediated cell death. Furthermore safety evaluation was done in HEK-293 cells and CF rat. Repeated dose toxicity study was carried out to find the possible toxic effect of SIE. In this study no significant change in body weight, food and water intake, behaviour, or mortality was observed as compared with control group. No significant changes were observed in organ weight and macroscopic parameters of vital organs when compared with control group.

In present study, biochemical parameters varied widely between different dose groups and sex, but these changes were not significant and altered values fall within normal ranges. Changed biochemical parameters do not show a dose response. Liver function was evaluated by using ALT, AST, ALP, and total bilirubin level, because they are liver function markers. In this study no significant change was observed in the level of liver function markers. The serum levels of triglycerides (TG), total cholesterol (TCHO), total protein (TP), albumin (ALB), total Glucose (GLU), calcium (Ca), and inorganic phosphorus (IP) were assessed to find out the general metabolic changes. Their values show no significant change and linear profile in various groups. CRTEA, BUN, and UREA level were observed to evaluate the kidney function. They also show no significant difference and dose response [103].

All haematological parameters lie within normal range and did not show any significant changes between different dose groups and sex. These results suggested that SIE does not produce any adverse effect on blood under these experimental conditions. The present toxicological study suggests that SIE does not show signs of toxicity and safer to be used as an alternate therapeutic agent.

Mass spectrometry method was used to detect the chemical constituent of SIE. Further research studies are still required to find out the mechanism of action of specific bioactive compounds responsible for antibreast cancer activity. Phenol and other bioactive compounds present in SIE are responsible for higher radical scavenger activity. Result of the present study shows that these bioactive components could exert anticancer activity due to their antioxidant potential; as well as they are also involved in modulation of signalling pathways.

## 5. Conclusion

Together, the findings of* in vitro* cytotoxicity on normal cell line and* in vivo* repeated dose toxicity study shows that SIE does not induce significant toxicity. SIE also show a potent* in vitro* antioxidant and antitumor activity. Antibreast cancer, antioxidant and toxicological evaluation of* Saraca indica* bark extract are promising and indicate that this herbal preparation may have a potential to be used in complementary and alternative medicine for breast cancer therapy.

## Figures and Tables

**Table 1:** Observation for toxicity symptoms as defined in the Common Toxicity Criteria. Animals were observed daily for sign of toxicity.

	Observation	Control group		Test groups (500 mg/Kg B.Wt., 1000 mg/Kg B.Wt., 1500 mg/Kg B.Wt. and 2000 mg/Kg B.Wt.)	
		Male	Female	Male	Female
(1)	Skin and fur	Normal	Normal	Normal	Normal
(2)	Eyes	Normal	Normal	Normal	Normal
(3)	Mucous membrane	Normal	Normal	Normal	Normal
(4)	Behavioural patterns	Normal	Normal	Normal	Normal
(5)	Salivation	Normal	Normal	Normal	Normal
(6)	Lethargy	Normal	Normal	Normal	Normal
(7)	Sleep	Normal	Normal	Normal	Normal
(8)	Diarrhea	Normal	Normal	Normal	Normal
(9)	Coma	N.O.	N.O.	N.O.	N.O.
(10)	Tremors	N.O.	N.O.	N.O.	N.O.
(11)	Vomiting and hematemesis (Vomiting Blood)	N.O.	N.O.	N.O.	N.O.

N.O. = Not observed.

**Table 2:** Result of serum biochemical analysis on day 15 of oral gavage administration of 500, 1000, 1500, and 2000 mg/Kg B.Wt. of SIE to male CF rats. NS represents the no statistical differences, when the test groups were compared to the control group.

	Parameter	Control group		500 mg/Kg B.Wt.		1000 mg/Kg B.Wt.		1500 mg/Kg B.Wt.		2000 mg/Kg B.Wt.		*P* value
		Mean	±SD	Mean	±SD	Mean	±SD	Mean	±SD	Mean	±SD	
1	**UREA**	**29.36**	5.128	** 35.62**	3.858	**27.96**	12.726	** 28.88**	4.604	** 28.1**	3.350	NS
2	**ALT**	**63.88**	15.535	** 88.1**	17.623	** 62.9**	22.121	** 68.04**	20.325	** 74.55**	16.465	NS
3	**AST**	**227.1**	41.620	** 274.56**	41.891	** 219.08**	81.350	**202.78**	23.720	** 219.075**	25.545	NS
4	**ALP**	**730.9**	108.3	**750.4**	103.00	** 737.10**	160.9	**719.5**	133.60	**727.30**	52.16	NS
5	**TG**	**48.44**	13.195	** 51.26**	17.018	** 53.2**	22.097	** 53.48**	19.278	**60.325**	17.986	NS
6	**TCHO**	**69.10**	7.69	**72.56**	11.121	**68.58**	7.43	** 73.22**	15.64	** 71.01**	13.89	NS
7	**TP**	**7.438**	0.863	**7.504**	0.671	**6.602**	0.886	** 7.252**	0.524	** 7.63**	0.843	NS
8	**ALB**	**3.364**	0.403	**3.638**	0.258	**3.162**	0.449	** 3.444**	0.246	** 3.74**	0.371	NS
9	**GLU**	**124.22**	44.408	** 142.94**	70.161	** 135.08**	29.697	**139.14**	10.95	** 136**	60.352	NS
10	**Ca**	**9.716**	1.267	** 10.356**	1.166	**8.882**	1.315	**10.092**	0.798	**10.357**	1.243	NS
11	**IP**	**7.564**	1.131	**11.2275**	2.230	**9.448**	2.222	** 9.386**	0.951	** 9.46**	1.155	NS
12	**TBIL**	**0.114**	0.020	** 0.170**	0.055	**0.184**	0.073	**0.16**	0.021	** 0.18**	0.048	NS
13	**CREA**	**0.524**	0.089	**0.546**	0.052	**0.464**	0.112	** 0.492**	0.014	** 0.527**	0.021	NS
14	**BUN**	**13.686**	2.391	** 16.982**	1.833	**12.434**	2.180	**13.468**	2.143	**13.092**	1.556	NS

**Table 3:** Result of serum biochemical analysis on day 15 of oral gavage administration of 500, 1000, 1500, and 2000 mg/Kg B.Wt. of SIE to female CF rats. NS represents the no statistical differences, when the test groups were compared to the control group.

	Parameter	Control group		500 mg/Kg B.Wt.		1000 mg/Kg B.Wt.		1500 mg/Kg B.Wt.		2000 mg/Kg B.Wt.		*P* value
		Mean	±SD	Mean	±SD	Mean	±SD	Mean	±SD	Mean	±SD	
1	**UREA**	**27.6**	2.094	**37.48**	5.53	**34.6**	9.361	**27.92**	2.172	**31.02**	5.005	NS
2	**ALT**	**69.4**	14.049	**87.64**	27.677	**72.22**	11.336	**65.44**	7.158	**58.62**	8.545	NS
3	**AST**	**227.76**	18.709	** 249.92**	20.422	**273.9**	23.13	**233.68**	37.570	**222.28**	38.749	NS
4	**ALP**	**785.3**	107.7	** 725.60**	187.7	**728.80**	46.32	**711.80**	102.20	**741.6**	117.10	NS
5	**TG**	**35.56**	9.916	**38.48**	10.185	**42.24**	9.333	**39.3**	3.903	**46.26**	11.007	NS
6	**TCHO**	**58.9**	10.342	**62.9**	6.264	**65.32**	27.640	**60.38**	7.960	**55.36**	11.083	NS
7	**TP**	**6.958**	0.370	**7.558**	0.585	**7.722**	1.865	**7.41**	0.208	**7.344**	0.368	NS
8	**ALB**	**3.358**	0.138	**3.574**	0.277	**3.84**	30.315	**3.518**	0.216	**3.528**	0.207	NS
9	**GLU**	**144.56**	32.497	**167.16**	98.128	**97.12**	50.640	**95.92**	33.358	**134.64**	28.068	NS
10	**Ca**	**9.958**	1.306	**9.928**	1.511	**10.818**	0.484	**10.206**	0.579	**10.414**	0.896	NS
11	**IP**	**9.06**	1.593	**10.922**	1.766	**10.054**	4.495	**8.61**	0.993	**8.758**	1.175	NS
12	**TBIL**	**0.15**	0.034	**0.15**	0.024	**0.14**	0.171	**0.148**	0.033	**0.17**	0.041	NS
13	**CREA**	**0.495**	0.020	**0.612**	0.057	**0.548**	0.045	**0.51**	0.064	**0.528**	0.068	NS
14	**BUN**	**12.86**	0.970	**17.470**	2.587	**14.700**	3.449	**13.004**	1.017	**14.518**	2.302	NS

Urea, alanine aminotransferase (ALT), aspartate aminotransferase (AST), alkaline phosphatase (ALP), triglycerides (TG), total cholesterol (TCHO), total protein (TP), albumin (ALB), total glucose (GLU), calcium (Ca), inorganic phosphorus (IP), total bilirubin (TBIL), creatinine (CREA), and blood urea nitrogen (BUN).

**Table 4:** Haematological results on day 15 of oral gavage administration of 500, 1000, 1500, and 2000 mg/Kg B.Wt. of SIE to male CF rats. NS represent no statistical significant differences, when the test groups were compared to the control group.

	Parameter	Control group	500 mg/Kg B.Wt.	1000 mg/Kg B.Wt.	1500 mg/Kg B.Wt.	2000 mg/Kg B.Wt.	*P* value
		Mean ± SD	Mean ± SD	Mean ± SD	Mean ± SD	Mean ± SD	
1	Hgb (g/dL)	13.03 ± 0.44	13.08 ± 0.259	13.28 ± 0.43	13.00 ± 0.58	13.02 ± 0.40	NS
2	T-RBC (×10^6^/mm^3^)	6.80 ± 0.08	6.71 ± 0.37	6.84 ± 0.41	6.73 ± 0.37	6.37 ± 0.42	NS
3	MCV (micron^3^)	57.55 ± 0.96	58.36 ± 1.20	57.00 ± 1.05	58.02 ± 2.42	57.62 ± 0.54	NS
4	HCT (%)	39.00 ± 0.60	39.94 ± 1.72	39.52 ± 0.82	39.00 ± 1.30	39.06 ± 0.27	NS
5	MCH (pg)	19.23 ± 0.76	19.64 ± 0.59	19.48 ± 1.29	19.38 ± 1.00	19.20 ± 0.63	NS
6	MCHC (g/dL)	33.78 ± 0.93	33.32 ± 0.51	33.64 ± 0.80	33.40 ± 1.07	33.22 ± 0.94	NS
7	WBC ×10^3^	4.30 ± 1.05	4.91 ± 1.25	4.87 ± 0.81	4.59 ± 0.97	4.47 ± 0.47	NS
8	RDW	10.60 ± 0.39	10.72 ± 0.16	10.90 ± 0.42	10.86 ± 0.54	10.56 ± 0.33	NS
9	MPV	4.47 ± 0.45	4.36 ± 0.40	4.08 ± 0.26	4.70 ± 0.26	4.47 ± 0.31	NS
10	PLT (×10^3^/mm^3^)	756.3 ± 40.15	710.2 ± 67.06	761.0 ± 54.66	739.2 ± 26.29	736.6 ± 56.06	NS

**Table 5:** Haematological results on day 15 of oral gavage administration of 500, 1000, 1500, and 2000 mg/Kg B.Wt. of SIE to female CF rats. NS represent no statistical significant differences, when the test groups were compared to the control group.

	Parameter	Control group	500 mg/Kg B.Wt.	1000 mg/Kg B.Wt.	1500 mg/Kg B.Wt.	2000 mg/Kg B.Wt.	*P* value
		Mean ± SD	Mean ± SD	Mean ± SD	Mean ± SD	Mean ± SD	
1	Hgb (g/dL)	12.63 ± 0.50	12.68 ± 0.66	13.16 ± 0.38	13.16 ± 1.00	12.80 ± 0.80	NS
2	T-RBC (×10^6^/mm^3^)	6.76 ± 0.26	5.99 ± 0.49	5.99 ± 0.49	6.72 ± 0.49	6.44 ± 0.82	NS
3	MCV (micron^3^)	56.90 ± 2.10	57.08 ± 1.56	57.42 ± 1.78	54.75 ± 1.81	55.02 ± 1.77	NS
4	HCT (%)	38.47 ± 0.37	34.23 ± 2.06	34.40 ± 2.73	36.80 ± 2.88	35.35 ± 3.30	NS
5	MCH (pg)	20.33 ± 1.25	21.15 ± 0.37	22.12 ± 1.42	19.60 ± 1.10	20.00 ± 1.64	NS
6	MCHC (g/dL)	35.67 ± 1.01	37.08 ± 0.41	38.48 ± 1.94	35.85 ± 1.00	36.32 ± 2.24	NS
7	WBC ×10^3^	5.35 ± 1.03	5.07 ± 0.76	4.88 ± 1.22	5.08 ± 1.95	5.32 ± 1.05	NS
8	RDW	8.87 ± 1.32	10.45 ± 0.33	9.40 ± 0.96	8.67 ± 1.02	10.81 ± 1.42	NS
9	MPV	4.00 ± 0.36	4.02 ± 0.33	4.26 ± 0.09	4.45 ± 0.13	4.24 ± 0.09	NS
10	PLT (×10^3^/mm^3^)	736.3 ± 47.20	759.0 ± 67.56	750.8 ± 84.40	796.8 ± 39.34	742.4 ± 74.48	NS

**Table 6:** Organ weight as a percent of total body weight in male CF rats. NS signifies no statistical differences when the test groups were compared to the control group.

	Control group		500 mg/Kg B.Wt.		1000 mg/Kg B.Wt.		1500 mg/Kg B.Wt.		2000 mg/Kg B.Wt.		*P* value
	Mean	±S.D.	Mean	±S.D.	Mean	±S.D.	Mean	±S.D.	Mean	±S.D.	
Adrenal Rt	**0.0116**	0.0036	**0.0118**	0.0017	**0.0116**	0.0031	**0.0114**	0.001	**0.0114**	0.0018	NS
Adrenal Lt	**0.0118**	0.0011	**0.0121**	0.0031	**0.0131**	0.0038	**0.0122**	0.0013	**0.0117**	0.0018	NS
Brain	**0.8174**	0.0544	**0.8957**	0.1333	**0.7797**	0.229	**0.8074**	0.0925	**0.7726**	0.1093	NS
Gonad Rt	**0.594**	0.039	**0.510**	0.052	**0.562**	0.065	**0.596**	0.081	**0.556**	0.136	NS
Gonad Lt	**0.594**	0.108	**0.513**	0.086	**0.557**	0.067	**0.666**	0.132	**0.497**	0.118	NS
Heart	**0.376**	0.025	**0.398**	0.059	**0.365**	0.046	**0.353**	0.057	**0.397**	0.011	NS
Kidney Rt	**0.395**	0.046	**0.408**	0.045	**0.374**	0.059	**0.412**	0.019	**0.390**	0.032	NS
Kidney Lt	**0.393**	0.051	**0.433**	0.061	**0.392**	0.06	**0.402**	0.017	**0.382**	0.028	NS
Liver	**3.903**	0.086	**3.962**	0.471	**3.891**	0.487	**3.923**	0.427	**3.623**	0.261	NS
Lungs	**0.888**	0.133	**0.816**	0.149	**0.803**	0.17	**0.901**	0.296	**0.937**	0.314	NS
Spleen	**0.420**	0.032	**0.368**	0.048	**0.437**	0.11	**0.447**	0.056	**0.433**	0.109	NS

**Table 7:** Organ weight as a percent of total body weight in female CF rats. NS signifies no statistical differences when the test groups were compared to the control group.

	Control group		500 mg/Kg B.Wt.		1000 mg/Kg B.Wt.		1500 mg/Kg B.Wt.		2000 mg/Kg B.Wt.		*P* value
	Mean	±S.D.	Mean	±S.D.	Mean	±S.D.	Mean	±S.D.	Mean	±S.D.	
Adrenal Rt	** 0.0098**	0.0008	**0.0091**	0.0022	**0.0103**	0.0031	**0.0103**	0.0004	**0.0106**	0.0051	NS
Adrenal Lt	**0.0098**	0.0008	**0.0106**	0.0008	**0.0115**	0.0017	**0.0107**	0.0012	**0.0094**	0.0029	NS
Brain	** 0.8104**	0.0492	**0.8067**	0.1106	**0.8831**	0.0506	**0.9363**	0.0803	**0.8745**	0.1116	NS
Ovary	**0.2493**	0.0356	**0.2306**	0.0851	**0.2440**	0.0830	**0.2489**	0.0846	**0.2942**	0.1196	NS
Heart	**0.3448**	0.0448	**0.3556**	0.0390	**0.3661**	0.0284	**0.3806**	0.0396	**0.4397**	0.1264	NS
Kidney Rt	**0.3532**	0.0254	**0.4152**	0.0233	**0.3720**	0.0374	**0.3681**	0.0298	**0.3850**	0.732	NS
Kidney Lt	**0.3554**	0.0380	**0.3868**	0.0188	**0.3812**	0.0305	**0.3671**	0.0458	**0.3800**	0.0894	NS
Liver	**3.5225**	0.2182	**3.6998**	1.8581	**4.1618**	0.5616	**3.9423**	0.3844	**4.1062**	0.8524	NS
Lungs	**0.7011**	0.0921	**0.7950**	0.1255	**0.6890**	0.0471	**0.9233**	0.3077	**0.8775**	0.3266	NS
Spleen	**0.4444**	1.4656	**0.4913**	0.7041	**0.4298**	0.1042	**0.4912**	0.7619	**0.4962**	0.8000	NS

**Table 8:** Inhibition of cell proliferation in terms of IC_50_ (*μ*g/mL) in different cell lines with 72 hours of treatment, data represented in mean ± SE.

Extract	MDAMB-231	MCF-7	HEK-293
SIE	128 ± 0.914	73.6 ± 0.625	>200

**Table 9:** Summary of compounds found in extract of SIE.

S. No.	Compound	Mol. Wt.
01	2-Methylbutanal oxime	101
02	Catechol	110
03	Uracil	112
04	Phenyl ethylamine	122
05	Protocatechuic acid	154
06	Gallic acid	170
07	Catechol derivative	190
08	Beta guanine	204
09	Epiafzelechin	274
10	Indolylmethyl glucosinolate	283
11	Catechin	290
12	Quercetin	302
13	Trimethyl apigenin	312
14	Tyramine beta xanthine	330
15	Gallic acid hexoside	332
16	Quercetin derivative	347
17	Ficochone A	348
18	Catechin derivative	352
19	Quercetin derivative	358
20	16-Methoxy tabersonine	367
21	Beta sitosterol	414
22	Hypophyllanthin	430
23	Phloridzin	437
24	Epicatechin	442
25	Quercetin-3-rhamnoside	448
26	Catechin derivative	458
27	Lignan	464
28	Galloyl-isorhamnetin	468
29	Myoinositol	492
30	Cellotriose	504
31	17-Decarboxy betanin	506
32	Nudiposide	552
33	Afzelechin	563
34	Lyoniside	576
35	Procyanidin	578
36	Catechin glucoside	584
37	Catechin derivative	598
38	Violaxanthin	600
39	Neohesperidin	610
40	Isorhamnetin	640
41	Tannin	724
42	Dicatechin gallate	730
43	Catechin derivative	741
44	Cyanidin	748

## Abbreviations

SIE: * Saraca indica* bark extract
ALT: Alanine aminotransferase
AST: Aspartate aminotransferase
ALP: Alkaline phosphatase
TG: Triglycerides
TCHO: Total cholesterol
TP: Total protein
ALB: Albumin
GLU: Total glucose
Ca: Calcium
IP: Inorganic phosphorus
TBIL: Total bilirubin
CREA: Creatinine
BUN: Blood urea nitrogen
Abs: Absorbance.
